# Prediction of Amyloidogenic and Disordered Regions in Protein Chains

**DOI:** 10.1371/journal.pcbi.0020177

**Published:** 2006-12-29

**Authors:** Oxana V Galzitskaya, Sergiy O Garbuzynskiy, Michail Yurievich Lobanov

**Affiliations:** Institute of Protein Research, Russian Academy of Sciences, Pushchino, Moscow Region, Russia; Harvard University, United States of America

## Abstract

The determination of factors that influence protein conformational changes is very important for the identification of potentially amyloidogenic and disordered regions in polypeptide chains. In our work we introduce a new parameter, mean packing density, to detect both amyloidogenic and disordered regions in a protein sequence. It has been shown that regions with strong expected packing density are responsible for amyloid formation. Our predictions are consistent with known disease-related amyloidogenic regions for eight of 12 amyloid-forming proteins and peptides in which the positions of amyloidogenic regions have been revealed experimentally. Our findings support the concept that the mechanism of amyloid fibril formation is similar for different peptides and proteins. Moreover, we have demonstrated that regions with weak expected packing density are responsible for the appearance of disordered regions. Our method has been tested on datasets of globular proteins and long disordered protein segments, and it shows improved performance over other widely used methods. Thus, we demonstrate that the expected packing density is a useful value with which one can predict both intrinsically disordered and amyloidogenic regions of a protein based on sequence alone. Our results are important for understanding the structural characteristics of protein folding and misfolding.

## Synopsis

Protein folding is one of the most challenging issues in biophysical science. During the past few years it has been shown that some diseases are connected with protein misfolding and the formation of insoluble aggregates called amyloid plaques. These processes may be associated with several diseases such as Alzheimer disease, Parkinson disease, Creutzfeldt-Jacob disease, and even certain forms of cancer. It has been shown that proteins with intrinsically disordered regions are involved in protein–protein or protein–nucleic acid interactions. The main objective of this paper is to report insights into the molecular mechanisms of amyloid aggregation. This has been done using the parameter of the observed number of contacts for each amino acid residue in globular state, further called expected packing density. By analysis of sequences alone, the authors have demonstrated that regions that possess strong expected packing density can be responsible for amyloidogenic properties of a protein, while regions with weak expected packing density correspond to disordered regions. A new concept is proposed that could aid in understanding protein folding, misfolding, and amyloidosis. The results help to explain that the nature of the amyloidogenic propensity of proteins is connected to their amino acid sequences that are able to form a large number of contacts.

## Introduction

Amyloid fibril formation is associated with an increase of β structure content, which leads to fibrillar aggregation [1]. However, it should be noted that an increased level of the beta structure is a characteristic property of several different types of protein aggregates (amyloid fibrils, amorphous aggregates) [2,3]. In addition to proteins observed in amyloid diseases, recent studies have shown that diverse proteins not related to any amyloid disease can aggregate into fibrils under destabilizing conditions [4−6]. Normal proteins can become toxic when they undergo fibrillation [7]. There is no consensus about toxicity of the different states: small oligomers, large oligomers, protofilaments, protofibrils, filaments, mature fibrils, or amorphous aggregates. Significant advancements in recent research have led to the discovery that the toxic species in the amyloid diseases may not be the fibrils themselves, but rather the pre-fibrillar aggregates [7]. A possible mechanism for toxicity of α-synuclein protofibrils has been demonstrated [8]. It has been shown that protofibrils can form elliptical pores, like bacterial toxins, which can puncture cell membranes, resulting in cell death [8]. Therefore, the mechanism of amyloid formation is under intensive investigation. Recognition of the factors that influence protein conformational changes and misfolding is one of the general fundamental problems, the solution to which will help us find effective treatments for amyloid illnesses.

The experimental observation that not all proteins are amyloidogenic (or at least that some proteins are less amyloidogenic than others) and that specific continuous regions of amyloid-forming proteins are more amyloidogenic than others suggests that there is a sequence propensity for amyloid formation. Moreover, the observation that some short peptides also can form amyloids implies that these segments, which usually are exposed to the environment, can nucleate the transition of native proteins into the amyloid state, and suggests that fibril formation is sequence-specific [9]. In the mechanism of amyloidogenesis for natively folded proteins such as β_2_-microglobulin and transthyretin, the partial unfolding observed is believed to be a prerequisite for the proteins' assembly into amyloid fibrils both in vitro and in vivo [10]. It has been suggested that residues with enhanced flexibility and solvent accessibility are important for the initiation of fibrillation [11]. This means that partial unfolding of the rigid native structure can provide a specific interface for the beginning of fibrillation. Thus, to understand the molecular mechanism of amyloidosis, it is necessary to find factors that induce partial unfolding of proteins and subsequent amyloid fibril formation at or near physiological conditions.

Some intrinsically disordered proteins are involved in amyloid diseases (type II diabetes, Alzheimer disease, and Parkinson disease). This fact may indicate that disorder is a necessary condition for aggregation. It has been shown that a very small change in the environment of such proteins often might cause their partial folding and aggregation [12]. Knowledge of characteristics that control the process of amyloid fibril formation is important for finding effective drugs for treatment of amyloid diseases.

Uversky and Fink in their review [13] illustrate that protein fibrillogenesis requires a partially folded conformation (originated from partial unfolding of intrinsically structured proteins or partial folding of intrinsically disordered proteins).

The first high-resolution (1 Å) crystal of an amyloid fiber formed by a sequence-designed polypeptide has been obtained [14]. Recently, the atomic structure of the cross-β spine [15] for a seven-residue peptide segment from Sup35 (GNNQQNY) was determined. It is a double β sheet, in which each sheet is formed from parallel segments stacked in register. Side chains protruding from the two sheets form a dry, tightly self-complementing steric zipper that bonds the sheets. Within each sheet, every segment is bound to two neighboring segments through stacks of both backbone and side-chain hydrogen bonds.

There are several computational methods for predicting a protein's propensity for amyloid fibril formation. In the work of Fernandez et al. [16] it was shown that a concentration of such defects as insufficient shielding of hydrogen bonds from water attack might yield an aggregation-induced nucleus. But the analysis of these defects revealed that the extensive exposure of hydrogen bonds to water attack might be a necessary but not sufficient condition to imply a propensity for organized aggregation [16].

A computational algorithm has been suggested that detects the nonnative (hidden) β strand propensity of sequences by consideration of the relationships between protein local sequence and secondary structure in terms of tertiary contacts [17]. This algorithm detects sequences within the protein that are favorable for triggering amyloid fibril formation. It is worthwhile to emphasize here that both algorithms for prediction of amyloidogenic properties of polypeptide chains that are considered above can be applied only to those proteins for which the three-dimensional structure is known.

Based on the physico–chemical properties of β aggregation sequences and a computational algorithm, a model was developed for predicting the aggregation rate for a broad range of polypeptide chains [18]. The model identifies aggregation sites within a protein and predicts the parallel or antiparallel organization of β sheets in a fibril. It should be noted, however, that the overpredictions of aggregation sites were not analyzed statistically.

On the other hand, there is a method for the prediction of amyloidogenic regions from amino acid sequence alone [19]. After the experimental investigation of the amyloidogenic properties of a model six-residue peptide and its mutants, the authors obtained a six-residue amyloidogenic pattern (STVIIE) and used this pattern for the identification of amyloidogenic fragments in proteins [19]. This amyloidogenic pattern has been used to validate the premise that the amyloidogenicity of a protein is indeed localized in short protein stretches (amyloid stretch hypothesis [20]). It has been demonstrated that the conversion of a soluble, non-amyloidogenic protein (SH3 domain of α-spectrin) into an amyloidogenic-prone molecule can be triggered by a non-destabilizing six-residue amyloidogenic insertion in a particular structural environment.

Recently, a new method for identifying fibril-forming segments of proteins has been suggested [21]. This method is based on the threading of six-residue peptides through the known crystal structure of an amyloid fiber [15] formed by the peptide from Sup35. The putative prediction is accepted as a prediction if its energy evaluated with RosettaDesign (http://www.rosettacommons.org) is lower than the threshold energy.

It should be added that molecular dynamics can yield valuable information about the structural changes that arise at the atomic level upon the formation of amyloid fibrils [23−24], while such information is difficult to obtain experimentally.

Another interesting new method (named PASTA) is based on sequence-specific interaction energies between pairs of protein fragments calculated from statistical analysis of the native folds of globular proteins [22]. This algorithm correctly predicts the positions of most aggregation-prone portions of some polypeptide chains.

The formation of a sufficient number of interactions is necessary to compensate for the loss of conformational entropy during the protein folding process. Therefore, the structural uniqueness of native proteins is a result of the balance between the conformational entropy and the energy of residue interactions. It seems that disordered regions in a protein chain do not have a sufficient number of interactions to compensate for the loss of conformational entropy that results from the formation of a globular state. On the other hand, a large increase in the energy of interactions will lead to a loss of the unique structure because the strengthening of contact energy will speed up folding, but it is also likely to lead to erroneous folds (for example, to amyloid fibrils).

It has been suggested that the lack of a rigid globular structure under physiological conditions might represent a considerable functional advantage for intrinsically disordered proteins, as their large plasticity allows them to interact efficiently with several different targets, as compared with a folded protein with limited conformational flexibility [25−29]. It has been shown that disordered regions are involved in DNA binding and other types of molecular recognition [30]. A large portion of the sequences of intrinsically disordered proteins contain segments of low complexity and high predicted flexibility [31−38]. It also has been indicated that a combination of low overall hydrophobicity and a large net charge represent a structural feature of intrinsically disordered proteins in comparison with small globular proteins [39,40]. There are currently several widely used methods for prediction of disordered regions: GlobPlot [41], a simple propensity-based approach for evaluating the tendency of residues to be in a regular secondary structure; PONDR VL3H [37], which is able to distinguish experimentally verified disordered proteins from globular proteins by various machine learning approaches; DISOPRED [42], in which the definition of disorder is restrained to regions that are missing from X-ray structures but are specifically recognized by a support vector machine in the DISOPRED model; and IUPred [43], which assigns the order/disorder status to residues on the basis of their ability to form favorable pairwise contacts. We were the first to our knowledge who used the number of contacts per residue as a parameter to distinguish folded and intrinsically disordered proteins [44]. We have extended our method to predict disordered regions and have made comparisons with the above-mentioned methods [45]. It has been demonstrated that our method is the best among widely used methods for the sets of proteins considered here.

Despite considerable efforts to understand the mechanism, it is still unclear what is responsible for amyloidogenic and disordered regions. The goal of this work is to test our hypothesis about whether protein regions that possess expected strong packing density are responsible for the amyloidogenic properties of proteins, while regions with weak packing density simultaneously are responsible for the appearance of disordered regions. We introduce a new parameter, namely mean packing density (number of residues within the given distance from the considered residue), which enables the prediction of both amyloidogenic and intrinsically disordered regions from protein sequence. These findings support the concept that the occurrence of amyloidogenic and intrinsically disordered regions has a similar basis in different peptides and proteins.

## Results

### Observed Mean Packing Density for 20 Types of Amino Acid Residues and Expected Packing Density Profiles

To calculate the packing density observed in protein structures, we have constructed two databases of protein structures. The first one [45,46] includes proteins with sequence identity less than 80% (database 80%). The second database consists of proteins with sequence identity less than 25% (database 25%). The average packing density observed in protein structures (database 25%) for each of 20 types of amino acid residues is shown in Table 1. For database 80%, the 20 values were not identical but very similar (they can be found in [45]), so that the correlation coefficient between the two sets of values was as large as 99.95%. These values were considered to be the expected packing density for the residues in each protein or peptide sequence studied further. It is worth noting here that three aromatic residues (tryptophane, tyrosine, and phenylalanine) have the highest observed packing density among the 20 amino acids in both databases. Among the many parameters that have been proposed to promote amyloid fibril formation is the π-stacking of aromatic residues [47,48]. Many amyloidogenic regions of proteins have high content of aromatic residues. From experimental works [49,50], one can suggest that aromatic residues favor aggregation because of hydrophobicity, size, and intrinsic β sheet propensity rather than aromaticity. The specific nature of the side-chain interactions for each protein will drive the rate of fibril formation as well as the resulting stability.

**Table 1:**
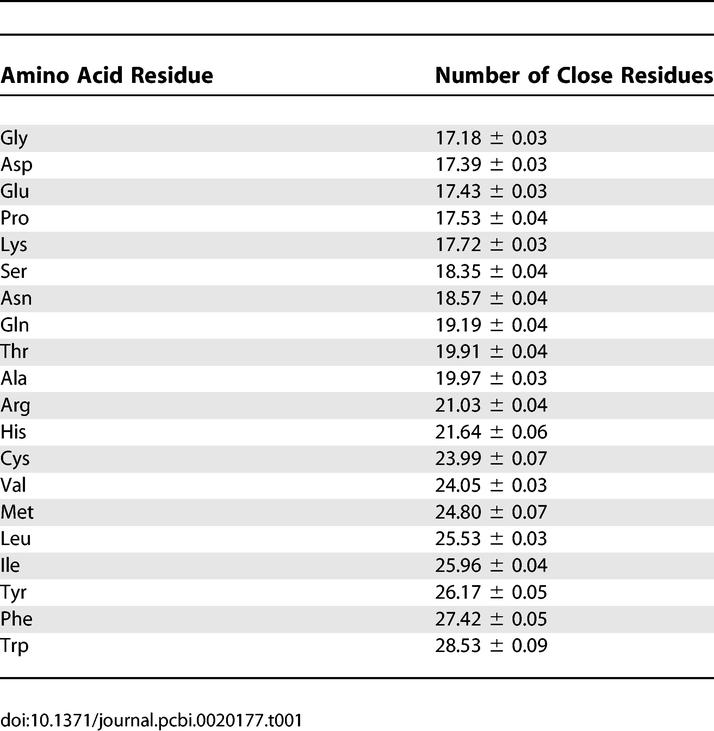
Mean Observed Packing Density for 20 Amino Acid Residues (and Errors in the Determination of the Average) Obtained Using a Contact Radius of 8.0 Å from Database 25%

The expected packing densities were averaged over a sliding window, and a packing density profile was produced (see Materials and Methods). Similarly, the other types of profiles were built using other scales instead of the scale from Table 1 (for example, hydrophobicity profile basing on hydrophobicity scale, etc.).

### Searching for Peptides That Are Fibril Formers and Fibril Nonformers

**Figure 1:**
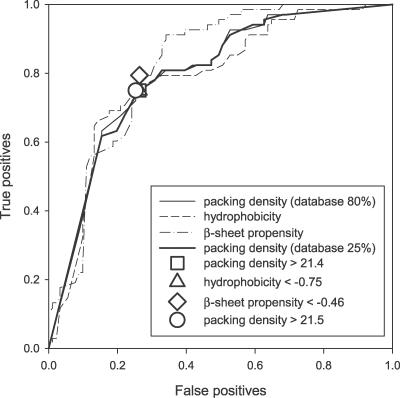
ROC Curves for Prediction of Amyloidogenic Regions in the Database of Fibril Formers and Fibril Non-Formers Peptides The symbols correspond to values chosen as thresholds.

To obtain a threshold for our predictions, we took a database of six-residue peptides, some of which were fibril formers and some of which were fibril nonformers [21]. The receiver operator characteristic (ROC) curves for our method are shown in Figure 1. The four ROC curves correspond to four scales: packing density for database 25% (Table 1), packing density for database 80% [46], hydrophobicity [51], and β sheet propensity [52]. For further investigations, we considered the following values the thresholds for predicting amyloidogenic regions (which gave rather a high level of true predictions, about 80%, as well as a rather low level of false predictions, about 25%): packing density greater than 21.5 and 21.4 for the two scales obtained from database 25% and database 80%, correspondingly; hydrophobicity less than −0.75, and β sheet propensity less than −0.46 (the corresponding points on the ROC curves [Figure 1] are marked with symbols). It should be mentioned that when we consider the packing density scale for database 80%, the ROC curve is slightly better; the threshold is 21.4.

### Searching for Optimal-Residue Long Sliding Window for Prediction of Amyloidogenic Regions

We collected a database of all known proteins and peptides that are associated with amyloid diseases, and for which the position of amyloidogenic regions is now experimentally examined (see Table 2). Amyloids are elongated fibrils that bind the aromatic dyes Congo red and Thioflavin-T have a common cross-β X-ray diffraction pattern [53].

**Table 2:**
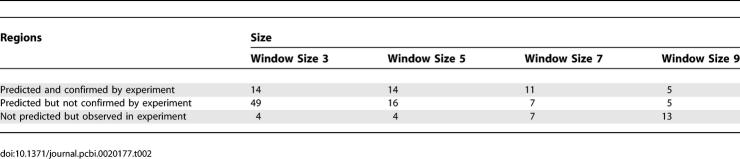
Comparison of Prediction of Amyloidogenic Regions Using Contact Density Scale and Varying Size of Sliding Window (Scale Obtained from Database 25%)

Varying the size of the sliding window (three, five, seven, and nine residues), we constructed a packing density profile for each of these proteins and peptides. We predicted a region as amyloidogenic if expected packing density for the region (with size equal or greater than size of the window) is above the considered threshold. Our hypothesis is that regions with strong expected packing density should correspond to aggregation regions, which presumably intersect with amyloidogenic regions of proteins. The number of predicted amyloidogenic regions are presented in Table 2. One can see that the window size of seven residues is optimal for the prediction of amyloidogenic regions. The result was very similar for the scale obtained from the 80% database (cutoff is 21.4 for this scale).

### Searching for Amyloidogenic Regions in Proteins with Known Disease-Related Regions

We constructed a packing density profile using a sliding window of seven residues for each of the proteins and peptides considered here. The experimentally observed amyloidogenic regions and the predicted ones are presented in Table 3 (25% database). One can see that for eight of 12 examined proteins and peptides the predictions are consistent with the experimentally found amyloidogenic regions.

**Table 3:**
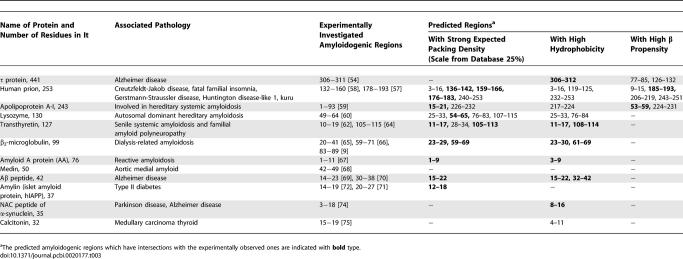
Predicted versus Experimentally Observed Amyloid-Forming Regions in Amyloidogenic Proteins and Peptides

In Alzheimer disease, τ-protein forms neurofibrillary tangles, which are bundles of paired helical filaments. A single region (amino acid residues 306−311), which is shown experimentally to be amyloidogenic [54], is correctly predicted by our method when we use a sliding window of five residues.

Despite a large body of experimental data related to the search for amyloidogenic regions in human prion protein, it is difficult to determine which regions these are. It has been shown that helix 1 (residues 144–153) of human prion protein (PrP) plays a critical role in the amyloidogenic process [55,56]. Peptides corresponding to three helical regions (residues 144−154, helical region one; residues 178–193, helical region two; and residues 198–218, helical region three) have been synthesized and studied [57]. The peptides corresponding to the second helical region, residues 180–193 and residues 178–193, are the only ones that form an amyloid structure, according to data obtained by electron microscopy and Congo red birefringence [57]. By using two intrinsic fluorescent variants of this protein (Y150W and F141W), conformational changes confined to the 132–160 segment were monitored [58]. Our predicted fragments intersect with all helices.

Most mutations described in apolipoprotein A (ApoA) are within the N-terminal portion of the protein (residues 1–93), which represents the proteolysis fragment that is incorporated into amyloid deposits [59]. We predict as amyloidogenic one region (residues 15–21) within the N-terminal portion as well as one additional region in the C-terminal part of apolipoprotein A, which has strong expected packing density.

The experimentally found amyloidogenic fragment of lysozyme (residues 49–64), which has been specifically implicated in amyloidogenic conversion [60,61], is a part of the β domain in the native structure of the protein. Our predictions for lysozyme are consistent with experimental results; however, three additional fragments (25–33, 76–82, and 107–114) are also predicted.

The most amyloidogenic peptide fragments from transthyretin (TTR) have been demonstrated in two regions: residues 10–19, which encompass the A strand of the inner β sheet structure that readily forms amyloid fibrils when dissolved in water at low pH [62,63]; and residues 105–115, which adopt an extended β strand conformation that is similar to that found in the native protein [64]. We predicted correctly these important regions (11–17 and 105–113) and one additional region with strong expected packing density.

It has been found experimentally that the following sequences play a dominant role in the amyloidogenesis of β_2_-microglobulin: residues 20–41 [65], residues 59–71 [66], and residues 83–89 [9]. All predicted regions are consistent with the experimental data except for fragment 83–89.

Reactive (or secondary) amyloidosis is characterized by the extracellular deposition of amyloid fibrils containing predominantly amyloid A protein (AA), which is a proteolytically derived fragment of serum amyloid A (SAA) protein. The N-terminus of amyloid A protein (residues 1–11 of AA protein) was shown to be the amyloidogenic part of the molecule [67]. We predicted this region correctly (residues 1–9).

Medin is the main constituent of the aortic medial amyloid. It is derived from a proteolytic fragment of lactadherin, a mammary epithelial cell–expressed glycoprotein that is secreted as part of the milk fat globule membrane. It was previously demonstrated that an octapeptide fragment of medin (residues 42–49, NFGSVQFV) forms typical well-ordered amyloid fibrils [68]. The last four residues (residues 47–50) have a large expected packing density, yet this region is not predicted by the rules of our algorithm (a region must be at least seven residues).

It has been shown that residues 16–20 in amyloid β (Aβ) peptide are essential for the peptide's polymerization [69]. Also, solid-state NMR and site-directed spin labeling experiments suggest that residues 30–38 [70] form a β strand in the fibrils. Our predictions (residues 15–22) are consistent with the first region.

It has been shown that a fragment (residues 20–27) from amylin (also called human islet amyloid protein or hIAPP) is amyloidogenic and cytotoxic [71]. Other than this one, the shortest active fragments capable of self-assembly were found to be pentapeptides FLVHS (residues 15–19) and NFLVH (residues 14–18) [72]. One of the fragments (residues 12–18) is correctly predicted by our method, but the second amyloidogenic region (residues 20–27) has expected packing density below the threshold.

Alpha-synuclein is a major component of Lewy bodies in Parkinson disease and is found to be associated with several other forms of dementia. The central fragment of α-synuclein (35 residues long), which has been isolated from purified amyloid of Alzheimer disease brains, [73] is called the non-Aβ component of Alzheimer disease amyloid (NAC). It has been shown that the N-terminal fragment of NAC (residues 3–18) forms aggregates and displays a transition from random coil to β sheet structure [74]. On the contrary, the C-terminal fragment of NAC (residues 19–35) remains in solution with random coil conformation under the same conditions [74]. No regions with expected packing density over 20.5 are observed. The predicted region (residues 9–13) appears only if the threshold is 20.3. Thus, we consider this prediction as a failure.

It has been shown that a peptide consisting of residues 15–19 of the human hormone calcitonin forms highly ordered fibrils, which are similar to those formed by the entire hormone sequence [75]. This region is not predicted by the rules of our algorithm.

Our predicted regions are consistent with known disease-related regions for eight of 12 experimentally well-studied amyloidogenic peptides and proteins (transthyretin, β_2_-microglobulin, lysozyme, prion protein, and others). This result strongly indicates that the aggregation capability of a protein chain is one of the common properties of amyloid fibrils. Moreover, it should be noted that regions with high packing density are often surrounded by amino acids that disrupt their amyloidogenic capability, regions with weak expected packing density, that is, amyloid breakers.

Here we also tested the ability of two other scales, hydrophobicity [51] and β sheet propensity [52], to predict amyloidogenic regions and compared these results with our method of expected packing density. The choice of the thresholds (Figure 1) for these predictions was made in the similar way. On the one hand, from 18 experimentally determined amyloidogenic regions, the expected packing density scale finds 14 regions (see Tables 2 and 4), while the hydrophobicity scale finds nine, and the β sheet propensity scale finds two regions (in other words, the packing density scale misses four amyloidogenic fragments while the hydrophobicity scale misses nine fragments and the β sheet propensity scale misses 16). On the other hand, the scale of expected packing density finds seven additional regions while the scale of hydrophobicity finds seven extra regions, and the scale of β sheet propensity finds six additional regions, the amyloidogenic role of which is not confirmed by experiment. Therefore, here we suggest a new property of peptides and proteins that can be used to predict the formation of amyloid fibrils: regions with strong expected packing density.

**Table 4:**
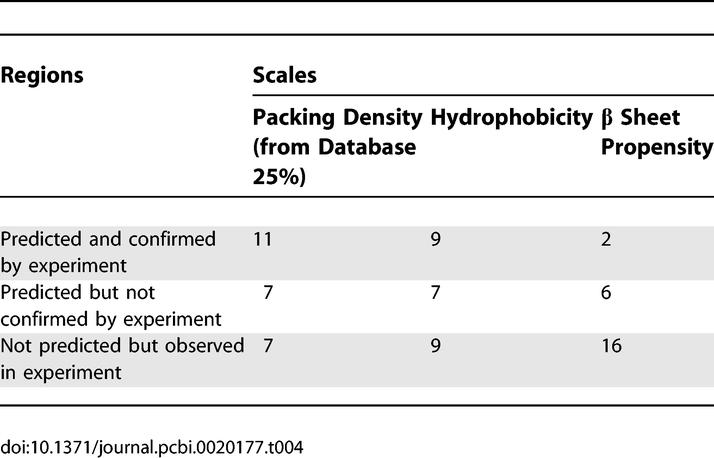
Comparison of Prediction of Amyloidogenic Regions Using Different Scales

### Searching for Intrinsically Disordered Regions

**Figure 2:**
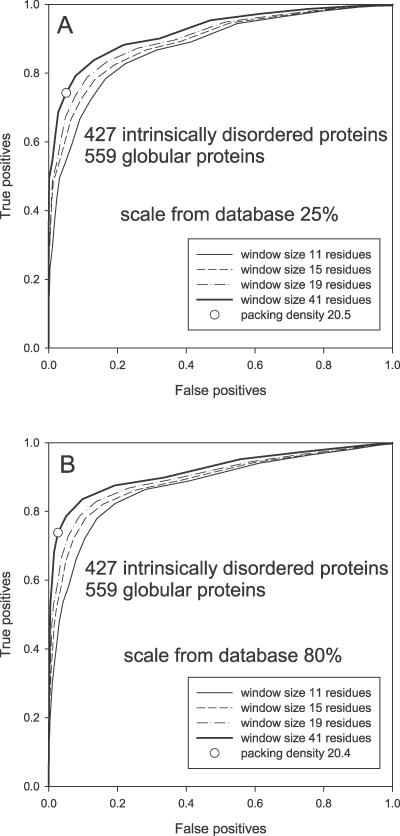
ROC Curves for Prediction of Intrinsically Disordered Regions Each ROC curve corresponds to predictions with specified (on the legend) size of the sliding window. The open circle corresponds to the value of packing density that is chosen as a threshold, 20.5 for database 25% (A) and 20.4 for database 80% (B).

To test the quality of our predictions of intrinsically disordered regions in proteins, we have used two databases, of which one has 427 intrinsically disordered proteins and regions [76] and the other has 559 fully folded proteins [43]. The ROC curves obtained with different sizes of the sliding window are shown in Figure 2. The best result corresponds to the case where we construct the packing density profile smoothed over the sliding window of 41 residues; we chose 20.4 (the corresponding point is marked as a large circle) as the threshold when we use the scale from database 80% (true positives 0.74 and false positives 0.03) and 20.5 when we use the scale from database 25% (true positives 0.74 and false positives 0.05).

To test the quality of predictions obtained by our method compared with other methods of prediction of disordered regions such as IUPred [43], DISOPRED2 [42], PONDR VL3H [37], and GlobPlot [41], we examined the same proteins that were used by Dosztanyi et al. [43], who compared the quality of predictions obtained by their method IUPred with DISOPRED, PONDR VL3H, and GlobPlot (the data on these methods were taken from [43]). These were a dataset of globular proteins (559 proteins) and long disordered protein segments (129 proteins). Table 5 demonstrates that our method (FoldUnfold) showed improved performance over these widely used methods on these sets of proteins (the averaging for our method is done in the same two ways as for the other methods [43]—over amino acid residues and over proteins).

**Table 5:**
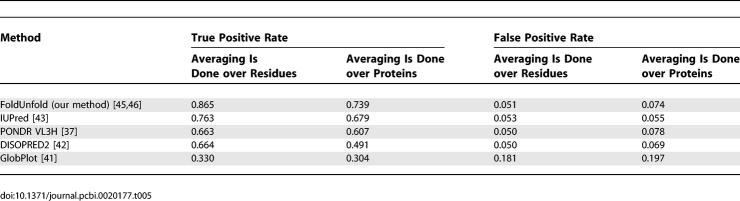
Performance of Disorder Prediction Methods on Datasets of Globular Proteins (559 Proteins) and Long Disordered Protein Segments (129 Proteins) [43] (Packing Density Scale Obtained from Database 25%)

## Discussion

**Figure 3:**
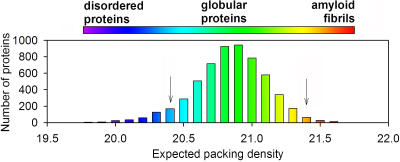
Histogram Representing the Distribution of 5,829 Globular Protein Domains (Database 80%) as a Function of the Expected Packing Density Arrows indicate upper and lower thresholds obtained from the ROC curves (see Figures 1 and 2) which correspond to unusually strong and unusually weak expected packing density.

We demonstrate that expected packing density is a useful value for the prediction of both intrinsically disordered and amyloidogenic regions of a protein based only on its sequence. In Figure 3, a distribution of average packing densities of globular proteins, is presented. The determined thresholds (21.4 for amyloidogenic regions and 20.4 for intrinsically disordered ones) correspond to the ends of this distribution.

Structures of peptides such as NNQQNY (derived from Sup35 protein [15]), KFFEAAAKKFFE (a designed 12-mer peptide [14]), and YTIAALLSPYS (derived from transthyretin [77]) confirm that the peptides adopt an extended β-strand conformation in amyloid fibrils. These fibrils achieve their stability through optimal values of main-chain and dihedral angles, as well as through extensive hydrophobic packing of side chains (hydrophobic template, Serrano's pattern—STVIIE) and salt bridge formation from polar side chains (polar template, Eisenberg's pattern—NNQQNY). It should be emphasized that between these two templates there probably exist many different intermediate variants. Our approach finds amyloidogenic regions closer to the hydrophobic template than to the polar one.

If amyloid fibril formation is a generic feature of proteins [5], some common properties of amino acid sequences possessing amyloidogenic propensities should be observed. Experimental data as well as theoretical analyses can help reveal the common structural and chemical properties for this process, one of which is the tight packing density.

We tried to collect all known amyloidogenic proteins and peptides for which disease-related regions are experimentally localized. By analysis of primary structure alone, we have demonstrated that regions that possess strong expected packing density can be responsible for the amyloidogenic properties of a protein, while regions with weak expected packing density correspond to disordered regions. A new concept is proposed that could aid in the understanding of protein folding, misfolding, and amyloidosis.

Our study provides new insights into the process of amyloid formation. The results help to explain that the nature of the amyloidogenic propensity of proteins is related to their amino-acid sequences that are able to form a large number of contacts. Our results can help determine the amyloidogenic propensity of amyloidogenic proteins for which the position of amyloidogenic regions now remains unexplored experimentally.

## Materials and Methods

### Observed packing density for 20 types of amino acid residues.

The set of protein structures used for calculation of the packing density observed in protein structures was obtained by inspection of the SCOP (Structural Classification of Proteins) [78] database 1.61 release (for database 80%) and 1.65 release (for database 25%). For the first database, 5,829 domains from four general classes (*a*–*d*) with less than 80% sequence identity values were found: 1,133 all-α proteins from class *a,* 1,644 all-β proteins from class *b,* 1,617 α/β proteins from class *c,* and 1,435 α + β proteins from class *d*. A total of 3,769 domains from four general classes (*a*–*d*) with less than 25% sequence identity values were found (database 25%): 794 all-α proteins from class *a,* 928 all-β proteins from class *b,* 1,089 α/β proteins from class *c,* and 958 α + β proteins from class *d*. The observed packing density for each amino acid residue from this database was calculated as the number of close residues (within the given distance). In our case a residue is considered close to the given residue if any pair of their heavy atoms is at distance of less than 8 Å. The neighboring residues bound with peptide bonds (which are close in any case) are not taken into account. The mean observed packing density for each of 20 types of amino acid residues is presented in Table 1. These 20 values were used for prediction of packing density from protein sequences, that is, the expected packing density (we consider the expected packing density of a residue equal to the mean observed packing density of the corresponding residue in a globular state).

### Calculation of the expected packing density profile.

It is worthwhile to emphasize that the order of the residues may play an important role in protein folding and may account for regions with weak and strong packing density in a protein structure. To predict such regions in a protein, we construct a profile of the expected packing density for the protein sequence. The calculations are based on a sliding window-averaging technique. For each peptide and protein, in the prediction of amyloidogenic regions the sliding window size is varied from three to nine residues while the sliding window size is 11 (or 41) residues in the case of intrinsically disordered regions prediction. The packing density profile is calculated as follows. First, the expected packing density is determined for each residue (see Table 1); then, these numbers are averaged for five residues inside the window and assigned to the central residue of the window. Therefore, the influence of residues along the sequence flanking each window is included in our calculation. The value of the average expected packing density for every position of the polypeptide chain provides the packing density profile. If more than five residues in a row have values over a specified threshold, this region is predicted to be amyloidogenic. On the other hand, any region having more than 11 (or 41) residues with values below a specified threshold is predicted to be intrinsically disordered.

### Databases used to test our method.

To evaluate the accuracy of, and confidence in, our method of predicting amyloidogenic regions, a database of 67 peptides that are six-residue fibril formers and 91 peptides that are six-residue fibril nonformers was used [21]. To test our method, we also used the amino acid sequences of 12 disease-related amyloidogenic proteins and peptides (for which the position of amyloidogenic regions is localized experimentally); the sequences were taken from the SWISS-PROT database [79] (http://us.expasy.org/sprot/). To test our method for predicting intrinsically disordered regions, we used three databases. Two of them were downloaded from the Database of Protein Disorder DISPROT [76]. The first one consists of sequences of 427 completely intrinsically disordered proteins and intrinsically disordered fragments. The second database contains 129 intrinsically disordered proteins. The third database consists of 559 globular proteins without intrinsically disordered fragments [43]. This database was constructed using Protein Data Bank (PDB) entries from the above work.

### Evaluation of the quality of predictions.

To obtain the quality of predictions and to determine thresholds, we calculated true positive and false positive rates and made so-called receiver operator characteristic (ROC) curves. In predictions of intrinsically disordered regions, the true positive rate was calculated as the fraction of residues predicted as intrinsically disordered over the intrinsically disordered set of residues; the false positive rate was the fraction of predicted intrinsically disordered residues over the set of folded residues. Similarly, in the case of six-residue peptides that were fibril formers, the true positive rate was calculated as the fraction of peptides predicted as fibril formers in the fibril formers set of peptides while the false positive rate was the fraction of peptides predicted as fibril formers in the fibril nonformers set of peptides.

### The other scales for prediction of amyloidogenic regions.

Using hydrophobicity and β sheet propensity scales, we predicted the amyloidogenic regions of the considered proteins and peptides and evaluated the obtained results in a similar way to how we predicted these regions using packing density scales. The hydrophobicity scale of 20 types of amino acid residues was taken from the work of Fauchere and Pliska [51]. The β sheet propensities of the 20 types of amino acid residues in an internal β sheet position were taken from the work of Minor and Kim [52]. The original hydrophobicity and β sheet propensity scales were taken with reversed sign since the most hydrophobic and β sheet–predisposed amino acid residues have the largest negative values.

## Abbreviations

Aβ: amyloid β peptide
NAC: non-Aβ component of Alzheimer disease amyloid
ROC: receiver operating characteristic
